# International Comparisons of Alcohol Consumption

**Published:** 2003

**Authors:** Kim Bloomfield, Tim Stockwell, Gerhard Gmel, Nina Rehn

**Affiliations:** Kim Bloomfield, Dr.P.H., is an associate professor in the Department of Health Promotion Research, University of Southern Denmark, Esbjerg, Denmark. Tim Stockwell, Ph.D., is a professor and director of the National Drug Research Institute, Curtin University, Perth, Australia. Gerhard Gmel, Ph.D., is co-director of research at the Swiss Institute for the Prevention of Alcohol and Drug Problems, Lausanne, Switzerland. Nina Rehn, M.A. Pol. Sc., is technical officer, Management of Substance Dependence at the World Health Organization, Geneva, Switzerland

**Keywords:** international AODR (alcohol and other drug related) problems, international differences, cultural patterns of drinking, research quality, alcohol quantity–frequency methods, measure of AOD (alcohol and other drug) volume and strength, cross-sectional study, gender differences, AOD abstinence, aggregate-level statistical data

## Abstract

International comparisons of alcohol consumption and its consequences can serve multiple purposes. For example, despite differences among countries in drinking cultures, drink sizes and strengths, and methods of measuring alcohol consumption, international survey research has provided a substantial amount of information on the rates of abstinence or current drinking, the frequency of drinking or binge drinking, and the mean consumption among both adults and youths in many countries. Other studies using aggregate-level data have analyzed per capita alcohol consumption in various countries. These studies can be used to relate per capita consumption to certain alcohol-related outcomes and to evaluate changes of both consumption and different outcomes within a country or across countries over time. Some problems associated with international research, however, such as issues of comparability of surveys, still need to be resolved.

Researchers in numerous countries have conducted analyses of alcohol consumption and general population surveys to ascertain the level and consequences of alcohol use. In recent years, investigators also have made attempts to compare drinking rates and other drinking variables across different countries. One reason for researching across national borders is the need for descriptive epidemiology (Room 1988). For example, national governments often want to know how their countries measure up against others in per capita consumption or in other comparative rankings of alcohol use. Another reason for comparative research is the desire to further theoretical knowledge; social scientists often employ comparative designs to develop or test theories. In the case of alcohol research, comparisons among different countries can help researchers determine how variations in social, cultural, political, environmental, and genetic factors can influence drinking behavior. For example, in the case of research on gender differences in alcohol use, international comparisons could help distinguish which differences in men’s and women’s drinking behavior can be attributed to biological differences and which to socio-cultural factors (Wilsnack et al. 2000).

Epidemiologic research into the underlying mechanisms (i.e., the etiology) of any disorder generally addresses two questions (Rose 1985):

- What are the causes of individual cases of the disorder (e.g., alcoholism)?
- What factors, such as sociocultural or political influences, contribute to the incidence of the condition in an entire population?

The type of question to be answered determines the level at which researchers compare data in international research. To answer the first question, investigators would mainly use individual-level studies (e.g., determine the level of alcohol consumption in individual drinkers within a country) to address within-population variability. To answer the second question, it may be more useful to conduct aggregate-level studies that determine overall alcohol consumption in a population (e.g., per capita consumption) to model between-population variability. Such aggregate-level analyses are particularly useful when some societal or environmental factors are constant or almost constant within a population. For example, Rose (1985) notes the hypothetical example of a population in which every person smokes 20 cigarettes per day. An epidemiologist who uses individual-level data to study risk factors for lung cancer in such a society could identify factors that vary between people with and without the disease (e.g., genetic susceptibility) but would not be able to identify smoking as a cause of lung cancer. Similarly, drinking patterns may show little variability within a drinking culture; accordingly, it may be more valuable to study the effects of various drinking patterns by comparing drinking patterns of different cultures. For any research question, however, both aggregate-level and individual-level studies have advantages as well as limitations (e.g., Greenland and Robins 1994).

This article describes some of the methodological problems involved in measuring drinking rates across countries, such as differences in drinking cultures, drink sizes, and measurement instruments. It then reviews the results of various types of studies that have examined drinking rates across countries. Finally, the article discusses how such results should be interpreted given the limitations of such studies, and it gives some recommendations for improving comparative international alcohol epidemiology for the future.

## General Methodological Issues in International Survey Research

### Differences in Drinking Cultures

Throughout the world, numerous different drinking cultures and attitudes toward alcohol exist. A more theoretical literature, which is somewhat separate from research comparing actual survey rates of alcohol consumption across countries, has been devoted to describing these differing drinking cultures. This research has had various historical traditions (Room 1988; Room and Mäkelä 2000), the most recent of which has focused primarily on differences between North American and European countries, dividing them into groups with either high or low per capita alcohol consumption, or so-called wet or dry cultures. These two categories also are commonly associated with various correlates, such as a history of temperance movements or a dominance of wine versus distilled spirits consumption (e.g., Peele 1997; Levine 1992; Room 1982).

Traditionally, the wet/dry distinction has been described as follows:

- In wet cultures, alcohol is integrated into daily life and activities (e.g., is consumed with meals) and is widely available and accessible. In these cultures, abstinence rates are low, and wine is largely the beverage of preference. European countries bordering the Mediterranean have traditionally exemplified wet cultures.
- In dry cultures, alcohol consumption is not as common during everyday activities (e.g., it is less frequently a part of meals) and access to alcohol is more restricted. Abstinence is more common, but when drinking occurs it is more likely to result in intoxication; moreover, wine consumption is less common. Examples of traditionally dry cultures include the Scandinavian countries, the United States, and Canada.

More recent comparative research, however, has found that, especially in Europe, the previous wet/dry division seems to be disappearing and a homogenization of consumption rates and beverage preferences is increasingly evident. For example, both Allamani and colleagues (2000) and Leifman (2001) have reported that wine consumption has decreased in the traditionally wet Mediterranean countries and that overall alcohol consumption has increased in the northern European countries. Room and Mäkelä (2000) also have reconsidered the simple wet/dry dichotomy and have instead proposed a new typology that considers a variety of drinking behaviors, such as the regularity of drinking and the extent of drunkenness. Such a typology may better fit the distinctions in drinking cultures that are emerging today. Nevertheless, the wet/dry dichotomy has represented a scale of extremes on which to measure drinking cultures and around which a fair amount of past research literature has been organized.

Researchers must take differences in drinking cultures into account when conducting international comparisons, in order to allow for a valid measurement of alcohol consumption in any given country. For example, in countries where drinking typically is frequent and regular, a simple questionnaire asking how often and how much people drink (i.e., a quantity–frequency index) may correctly measure most of the consumption. In countries where infrequent heavy episodic drinking occurs, however, questions regarding occasions when higher quantities of alcohol are consumed are indispensable. Some data collection instruments can be adjusted to the local drinking culture to accurately measure alcohol consumption and reflect the consumption patterns.

Because of the differences in drinking patterns, no single best instrument exists for measuring consumption. Nevertheless, a standard instrument that is flexible enough to cover most drinking patterns should be used in comparative research to ensure that the data obtained from different cultures are indeed comparable. In addition, the accuracy and comparability across studies of alcohol consumption measurements depend on several potential sources of measurement error. These include response rates among study participants, the mode of survey administration (e.g., face-to-face versus telephone interviews), and the representativeness of the sample. All of these factors can influence estimates of drinking variables. A recent international conference focused on developing consensus on questionnaire items for measuring alcohol consumption and alcohol-related social harm in international comparisons. The recommendations from this conference (Dawson and Room 2000), as well as the recently published *International Guide for Monitoring Alcohol Consumption and Related Harm* (World Health Organization [WHO] 2000), may help optimize comparability in international alcohol surveys.

The following sections demonstrate the complexity of assessing alcohol consumption using two examples—drink size and strength, and measurement instruments.

### Variation in Drink Sizes and Strengths

The basic problem for researchers conducting surveys of alcohol consumption is how to measure the amount of pure alcohol (chemically known as ethanol) a respondent consumes, both on an individual drinking occasion or day, and cumulatively over a longer period. All researchers conducting surveys of alcohol consumption must make assumptions about the serving size and alcohol content of the drinks people say they have consumed. In economically developed countries, however, it is becoming increasingly difficult to make such assumptions because of a bewildering array of beverages on the market that come in containers of varying sizes and with varying strengths. For example, in Australia at least 10,000 varieties of wine, distilled spirits, and fruit- or beer-based drinks are available for sale.

Another factor adding to the complexity of accurately measuring alcohol consumption is that consumption is conventionally expressed in grams of ethanol. One cannot ask respondents how many grams of ethanol they consume, however, because people generally do not think about their drinking in terms of alcohol content. Instead, researchers ask about the number of “drinks,” “units,” “bottles,” or “cans” a person typically consumes, depending on the national culture. These everyday units, which respondents recognize, contain varying amounts of ethanol, even within a particular country. This problem is further compounded by the fact that when drinks are poured from a common container (e.g., a bottle, cask, or can), the amounts poured will vary greatly both among “home measures” (Lemmens 1994) and among drinks served on licensed premises (Banwell 1999). For example, Kaskutas and Graves (2000) found that respondents in a sample of African American women commonly poured themselves “drinks” that contained up to six times the amount defined as a standard serving (i.e., 12 g alcohol). Similarly, Banwell (1999) reported that the average serving for a glass of wine in Melbourne bars was 180 mL, not 100 mL as defined both in surveys and by health promoters in Australia.

The WHO (2000) recommends that researchers address the issue of assumed drink sizes in order to enhance comparability among alcohol surveys in different countries. This approach requires that investigators in each country conduct observational studies to validate assumptions regarding typical serving sizes for each major beverage in different settings. Similarly, assumptions about typical drink strengths and container sizes, which ideally would be based on official sales data across time and place, should be validated. In addition, investigators could have respondents indicate the number of beverage containers or the portions of bottles consumed in cases where those measures could be more meaningful than the number of glasses.

Such local efforts to maximize the validity of the most basic (conceptual) unit of measurement in alcohol surveys—the “drink”—can improve the international comparability of alcohol surveys, provided that other methodological considerations, such as sampling methodology, are also addressed. With this approach, researchers in different countries may have to refer to differently sized “standard drinks” or drink containers because respondents most readily understand and report on these concepts. At the same time, however, investigators must have a valid estimate of the amount of ethanol present in each of these drinks or containers to ensure comparability of the data. For example, based on this information regarding drink size and alcohol content, four British pints of beer (16 g ethanol each), five North American bottles of beer (12–14 g ethanol each), and six Australian “middies” of regular beer (10 g ethanol each) would be considered to contain approximately the same quantity of ethanol (i.e., a total of approximately 60 g ethanol).

Researchers must also scrutinize assumptions about the usual ethanol content (the strength) of major beverages. For example, a recent Australian study noted substantial differences in the typical strengths of beers and distilled spirits across both time and place in Australia during the 1990s (Catalano et al. 2001). Similar findings were reported for wine in Canada (Single and Giesbrecht 1979). Furthermore, a study by the Finnish Foundation for Alcohol Studies (1977) documented changes in alcohol content as high as 6 percent for cider and 8 percent for distilled spirits in different countries over time.

### Methods for Measuring Consumption

The most commonly used and simplest measure of alcohol consumption is the quantity–frequency index. With this measure, respondents estimate how often they drink and how much they drink on a typical drinking occasion. One drawback of this approach is that the respondents tend to ignore occasional episodes of heavy consumption, which results in underestimates of true alcohol consumption (Gruenewald and Nephew 1994). However, if respondents are asked only about consumption on recent occasions—a so-called recent-recall approach—rather than average consumption, they generally report significantly higher amounts per day (see Lemmens et al. 1992).

Various examples of recent-recall approaches exist, such as drinking diaries or survey questions focusing on very recent alcohol consumption. These approaches appear to produce relatively high estimates of total alcohol consumption. Because the volume of alcohol reported in drinking surveys is normally only between 40 and 60 percent of the amounts known to be sold in the relevant region, researchers conducting drinking surveys generally assume that the higher the reported amounts are, the more accurately they reflect actual drinking behavior. Knibbe and Bloomfield (2001) compared the ability of six European national surveys to account for national levels of consumption as determined from sales data. The French survey, which contained only one question asking how much alcohol the respondents had consumed the day before the survey, achieved the highest estimate. In another analysis, the Australian Institute of Health and Welfare (Mathers et al. 1999) used a survey that inquired about alcohol consumption only over the 3 days prior to the survey. When the data were weighted for the day of the week, the reported consumption accounted for over 70 percent of the per capita alcohol consumption for that year. One plausible interpretation of these findings is that they indicate that poor recall is a major source of underestimation of alcohol consumption. Nevertheless, recent-recall approaches also have a major weakness in that they provide a valid picture only of recent behavior, which may not necessarily reflect typical consumption and may miss consumption by infrequent drinkers.

Another method for preventing underreporting of typical alcohol consumption is to use the graduated-frequency method. With this approach, respondents are asked to estimate how frequently they drink different daily quantities of alcohol—for example, on how many days per week or month they have 0, 1–2, 3–4, drinks, and so on. When researchers compared this method with both the quantity–frequency index and a weekly-recall method, the graduated-frequency method resulted in the highest estimates of alcohol consumption in a general population sample from Ontario, Canada (Rehm et al. 1999).

Several other methodological issues also must be addressed in order to maximize the comparability of different national alcohol surveys (WHO 2000). Although the choice of methods must reflect the major research questions being addressed, the main policy outcomes to be measured, and the resources available for the study, the WHO recommends that investigators pay attention to the following issues:

- The measures should reflect both the overall volume consumed and the patterns of drinking.
- Valid local estimates of typical units of alcohol consumption should be available.
- The study should include a reference time period that matches other measures used for harms, such as alcohol-related workplace problems in the past 12 months.
- Sampling methods should be designed to maximize the representativeness of samples.
- Whenever possible, researchers should use multiple methods to compare consumption internationally, using recent-recall, graduated-frequency, and per capita alcohol consumption data from each country studied.

The WHO (2000) recommends the graduated-frequency approach as the method of choice for most purposes; however, investigators have experienced difficulties in setting several comparable quantity thresholds across different countries. Given local differences in drink sizes and strengths, it is possible that only one or two such quantity thresholds, which should correspond to certain levels of risk of experiencing adverse alcohol effects, are practical for use in international comparative research. The WHO (2000) suggests that the thresholds for consumption that results in a medium risk of acute adverse outcomes should be set at > 60 g of alcohol^1^ per day for men and > 40 g of alcohol per day for women. For consumption that results in a high risk of adverse consequences, the thresholds should be > 100 g of alcohol per day for men and > 60 g of alcohol per day for women.

The use of different consumption thresholds for men and women—which are based on gender differences in alcohol metabolism—is still controversial. One review concluded that different thresholds are most appropriate when studying short-term and long-term physiological effects of alcohol (Graham et al. 1998). However, according to that review different thresholds are less important for the study of behavioral gender differences in the effects of alcohol consumption (e.g., alcohol-related injuries), which are moderated by the slower drinking pace frequently found in women, than for the study of biomedical gender differences (e.g., liver disease). Conversely, a recent case control study found marked gender differences in the risk of alcohol-related injury at different thresholds of consumption. In that study, women at all levels of alcohol consumption had a substantially greater risk of injury than men (Stockwell et al. 2002).

## International Survey Research

Numerous investigators have conducted studies of alcohol consumption among adults and youth in a number of countries. Such consumption data collected at the individual level (i.e., through interviews and questionnaires) have several advantages over aggregate-level data based on alcohol sales statistics. For example, individual-level studies can gather information on drinking patterns, such as average consumption, frequency of consumption, and other variables, such as heavy episodic drinking. Moreover, these studies allow for comparisons among subgroups in the population (e.g., based on age, gender, and ethnicity), which are not possible with per capita consumption data based on sales. The following sections review findings obtained in international surveys conducted among adults and youths.

### Surveys of Adults

Several international comparisons of drinking rates in adults based on general population surveys have been conducted in the last decade or so. The studies presented here meet the following criteria: (1) They are recent cross-sectional general population surveys. (2) They reported prevalence rates of drinking behaviors, such as rates of abstinence or current drinking (i.e., alcohol consumption in the past 12 months) and rates of heavy drinking, as well as mean alcohol consumption and mean frequency of drinking. Studies measuring alcohol dependence or alcohol problems are not discussed here. A comprehensive review of the literature to identify such studies was beyond the scope of this article. Rather, the authors chose studies they were familiar with and supplemented this information with the studies’ own literature and a confirmatory search of the Alcohol and Alcohol Problems Science Database (ETOH), which is maintained by the National Institute on Alcohol Abuse and Alcoholism (NIAAA), using such search terms as “international comparisons” and “cross-cultural comparisons.” Although this list of studies may not be exhaustive, it provides a general picture of the state of recent comparative alcohol epidemiology.

The studies reviewed here tend to represent bounded comparisons—that is, comparisons among several rather similar countries (Room 1988). Only two of the studies (Wilsnack et al. 2000; Fillmore et al. 1991) sought to include a wider array of countries from various regions (e.g., North America, Australia, the Middle East, and Europe). The other studies primarily included groups of member states of the European Union (EU) or Scandinavian countries. This selection reflects the present major funding bodies for such research, such as the EU. In addition, although such a selection may appear limited, a focus on relatively similar countries may improve the comparability of the data. Despite considerable variation in drinking cultures and research approaches, European countries tend to have more in common in these respects than do, for example, African and European countries (Room 1988).

With most of these studies, questionnaires (including discrepancies in translations) and response rates varied to such an extent that the resulting measurement and nonresponse bias discourage direct comparison of consumption levels (e.g., Simpura et al. in press; Knibbe and Bloomfield 2001). Thus, most studies also included other measures (e.g., gender ratios) to examine trends and relationships between drinking and other variables when comparing data across countries.

#### Abstinence

Several studies compared abstinence rates among adults in various countries. The countries included in the different studies vary, making an overall analysis difficult. The studies found the following results (see table 1):

- In investigating gender differences in drinking, the International Research Group on Gender and Alcohol conducted a comparison among men and women in 10 countries (Wilsnack et al. 2000). Using comparable measures constructed from the data sets collected in the study countries, the group found that lifetime abstention for both men and women was highest in Israel (ages 18–40 years) and lowest in the Czech Republic.
- Hupkens and colleagues (1993) analyzed data on beverage-specific drinking frequency from the Eurobarometer, an annual survey conducted in the EU to monitor the attitudes of the population toward the EU and its policies. The study reported that among the 12 EU member states in 1988, Ireland had the highest rate of abstinence (timeframe not specified) and Denmark had the lowest rate.
- In examining trends in alcohol use across as many as 15 European countries, Simpura and Karlsson (2001) reported rates of abstinence (definitions vary) for 1995. Among women, the rate of abstention was highest in Portugal and lowest in Denmark. Among men, the abstention rate was highest in Italy and lowest in Denmark.
- In a study examining episodic heavy drinking with a common questionnaire in four Scandinavian countries, Mäkelä and colleagues (2001) found that Sweden had the highest 12-month abstinence rate for men, Norway had the highest rate for women, and Denmark had the lowest rate for both genders.
- A recent EU project, funded partially by the European Commission, conducted a survey of alcohol use, alcohol-related problems, and attitudes toward alcohol in six European countries (Leifman 2002). Surprisingly, among these countries, abstinence in the last 12 months was lowest in Finland. Equally surprising, the highest rate of abstinence was found in France.

Overall, these studies indicate that in recent years the Mediterranean countries had comparatively higher rates of abstention than countries of central Europe. Furthermore, the abstinence rates in Scandinavian countries appear to be declining. Finally, Denmark appears as a special case in both Scandinavian and EU-wide comparisons as a country with low abstinence rates. These findings agree with the recently observed trend toward a homogenization of European drinking styles.

### Current Drinking

Two studies have looked at current drinking rather than abstinence in measuring the respondents’ drinking status. One study compared drinking patterns between Germany and the United States. This analysis found that within a comparable age range and with fairly similar instruments, Germany had one-third more current drinkers (i.e., people who had consumed alcohol in the past 12 months) than did the United States (Bloomfield et al. 2002). The other study was part of an EU concerted action that examined women’s alcohol consumption and alcohol problems (as well as gender differences in drinking patterns) in nine European countries through a secondary analysis of survey data. In one of its analyses of basic drinking measures, Ahlström and colleagues (2001) found that current drinking rates (i.e., consumption in the last 12 months) were highest among French men and Swedish women and were lowest among Italian men and women. These findings for current drinking (which is the inverse of abstinence) appear to fit the conclusions drawn regarding abstinence rates as described in the preceding section.

#### Frequency of Drinking

Another aspect of drinking behavior often measured in comparative studies is the frequency of drinking. Several studies have assessed this variable in international comparisons. These analyses, which included information from current drinkers only, had the following results:

- The study by Hupkens and colleagues (1993) involving 12 EU member states found that Spain had the highest frequency of drinking for men, Italy had the highest frequency for women, and Ireland had the lowest frequency for both genders (in general frequency categories^2^).
- Ahlström and colleagues (2001) determined that among nine European countries, France had the highest and Finland had the lowest frequency of drinking (i.e., number of drinking occasions within a month).
- Wilsnack and colleagues (2000), with their sample of 10 countries, found the highest frequencies of drinking (i.e., number of drinking occasions in a month) among Dutch women and Czech men and the lowest frequencies among Estonian women and men.

Two studies examined the rates of daily drinking in various countries. Leifman (2002) reported that when all respondents (i.e., drinkers and nondrinkers) were included in the analysis, Italy had the highest and Finland the lowest rates of daily drinking. Simpura and Karlsson (2001) examined seven countries regarding daily drinking; this analysis noted that wine countries had the highest rates, followed by beer-drinking countries and former spirits-consuming countries. This order was true for both men and women. Finally, in the two-country comparison of Bloomfield and colleagues (2002), respondents (i.e., drinkers and nondrinkers) in Germany reported almost twice as many drinking days as did U.S. respondents. All of these studies suggest that the main wine-consuming (and wine-producing) countries of Europe have the highest frequencies of drinking.

The international Collaborative Alcohol-Related Longitudinal Project (e.g., Fillmore et al. 1991) conducted meta-analyses on 39 longitudinal data sets^3^ on alcohol consumption from 15 countries. Its goal was not necessarily to examine differences in basic drinking rates across countries, but to examine and predict drinking patterns and problems over the life course (Fillmore et al. 1991). In one of its analyses examining the frequency of drinking, the study took into account national origin of the data sets. It grouped the studies into regions, including the United States, Canada (including New Zealand), Europe, and the United Kingdom (including Ireland). This comparison found that in relation to the United States (which was used as a reference), the frequency of consumption was highest in the United Kingdom, followed by the European countries and Canada (Johnstone et al. 1996).

#### Binge Drinking

Binge drinking (sometimes called heavy episodic drinking) has been included as a measure in some recent comparative studies:

- Wilsnack and colleagues (2000), in an analysis that included 10 countries, found that Canadian men and Swedish women had the highest percentages of drinkers who had engaged in heavy episodic drinking (the definitions of this term varied among the countries) in the last 12 months. Conversely, Israeli men and women had the lowest percentages of heavy episodic drinking. (This analysis included current drinkers only.)
- Studying data from four Scandinavian countries, and considering data from all respondents (drinkers and nondrinkers), Mäkelä and colleagues (2001) found that Danish men and women had the highest annual frequencies of consuming six or more drinks on one occasion, and Norwegian men and Finnish women had the lowest frequencies.
- In his study of six EU member states, Leifman (2002) reported that when both drinkers and nondrinkers were included in the analysis, people in the United Kingdom had the highest annual frequency of heavy drinking (defined as drinking a bottle of wine or the equivalent on one occasion), and people in France had the lowest.
- In a comparison between the United States and Germany including all respondents (Bloomfield et al. 2002), the number of days per month on which five or more drinks were consumed was almost twice as high in Germany as in the United States.

No consistent pattern in binge drinking rates emerges from these studies. This lack of consistency might be partially attributable to the small number of studies and the varying definitions of the behavior.

#### Mean Alcohol Consumption

Several recent international studies also have compared mean levels of alcohol consumption. In the study by Ahlström and colleagues (2001), Italian respondents reported the highest mean monthly consumption and Finnish respondents the lowest, when drinkers only were considered. Leifman (2002), who included all respondents in the analysis, found the highest mean annual alcohol consumption in the United Kingdom and the lowest consumption in Sweden. Mäkelä and colleagues (2001) reported that among the Scandinavian countries, Denmark had the highest and Norway had the lowest annual consumption in an analysis of all respondents. In the study comparing the United States and Germany (Bloomfield et al. 2002), mean monthly alcohol consumption among all respondents was more than twice as high among German respondents as among U.S. respondents. Finally, a somewhat older study examined differences in the sociodemographic correlates of drinking patterns in the general populations of Switzerland, Germany, and the Netherlands (Knibbe and Lemmens 1987). This study, which analyzed all respondents, found that Germans had the highest average weekly consumption in terms of standard glasses, followed by the Swiss and Dutch respondents.

As with binge drinking, these findings do not indicate a clear pattern in mean consumption among the countries studied. This lack of consistency could be accounted for by the varying methods of measurement used across the studies.

#### Summary

Overall, the studies reviewed in this section suggest that relatively consistent patterns appear to exist across the reported studies with respect to abstinence and frequency of drinking (e.g., relatively high abstinence rates in the Mediterranean countries and highest frequency of consumption in the wine-producing countries of Europe). Measures of binge drinking and mean consumption levels, however, exhibited less consistency. It is difficult to determine whether these inconsistencies stem from methodological problems or from real changes in drinking behaviors, which no longer fit the traditional typologies of drinking cultures (e.g., the wet/dry dichotomy). Such questions deserve increased attention as researchers develop methodology and concepts of future studies.

### Surveys of Youth

To date, researchers have conducted two large-scale international youth surveys relating to alcohol use, the Health Behavior of School-Aged Children (HBSC) survey and the European School Survey Project on Alcohol and Drugs (ESPAD). The HBSC, conducted for the fourth time in 1997–1998, included children ages 11, 13, and 15 in 26 European countries, Canada, and the United States (Currie et al. 2000). The ESPAD surveyed 15-year-olds from 30 European countries for the second time in 1999 (Hibell et al. 2000). The main advantage of both surveys is the use of a common methodology in all participating countries. For example, investigators in the participating countries of each survey used a common questionnaire, employed a standardized sampling methodology to ensure that their samples were representative of that country, surveyed the same age group(s) across countries, and collected their data in the same year. Moreover, both projects required minimum sample sizes as criteria for participation in the respective collaborative study, guaranteeing a certain statistical power, and thus precision, of the results. Finally, the surveys were conducted in school, a procedure that reduces nonresponse because usually only those students who are absent on the day of the survey do not participate (although responding to the questionnaire is voluntary). Thus, the response rates usually exceeded 85 percent. The data presented here refer to the most recent versions of both surveys.

#### Abstention Rates

The HBSC found that in all countries abstention from alcohol covaried highly between the sexes—that is, if the abstention rate was high among males, it usually was also high among females. In addition, the countries maintained their relative positions in abstention rates in all age groups tested. However, the differences among countries in abstention rates diminished among older students. The HBSC findings also suggested that with regard to abstention rates, the distinction between dry or wet drinking cultures either did not apply to adolescents or developed later in life. Thus, the HBSC found high abstention rates in prototypical wet countries, such as France and Switzerland, but also in Norway, the United States, and Israel. Conversely, in Denmark, Scotland, Wales, England, the Czech Republic, and Slovakia, abstention was uncommon even at age 13.

#### Weekly Drinking

The HBSC survey also provided data on the percentages of respondents who consume alcohol at least once a week. Because these percentages included the abstainers, the authors of this article recalculated the percentages of weekly drinkers relative to the percentages of drinkers to determine the proportion of weekly drinkers among all drinkers in each country. In general, these analyses demonstrate that as the percentage of drinkers increased, so did the percentage of weekly drinkers (r = 0.4).^4^ However, one group of countries diverged from this pattern by recording, among both males and females, high percentages of drinkers but low percentages of weekly drinkers. This group—which consisted of Poland, Lithuania, Estonia, Latvia, Finland, and Sweden—was the only group of geographically connected countries exhibiting a pattern that deviated from the patterns in other countries.

Countries with relatively low percentages of both drinkers and weekly drinkers included Norway, Switzerland, and the United States. Conversely, high percentages of both drinkers and weekly drinkers were found in England, Wales, Scotland, Denmark, and Greece. Again, no marked gender differences existed across the countries—if the percentage of male weekly drinkers was high, so was the percentage among females. Finally, the ranking of the countries with respect to weekly drinking remained stable among the different age groups tested, similar to what had been observed for the abstention rates. These findings in young people again did not seem to fit the prototypical wet/dry classification of adult drinking in that nations with high alcohol consumption among youth included both a wine country, Greece, and beer-drinking countries, such as Denmark, England, and Wales.

#### Drinking Patterns

The ESPAD survey, which included some countries not participating in the HBSC, allowed for more detailed analyses of drinking patterns. To this end, the authors of this article used the following variables from the 1999 ESPAD report (Hibell et al. 2000):

- Frequency of consuming different types of alcoholic beverages
- Alcohol consumption three times or more in the past 30 days
- “Binge” drinking (i.e., consumption of five or more drinks in a row) three times or more during the last 30 days
- Frequency of any alcohol consumption in the past 12 months, with abstinence defined as no alcohol consumption in the past 12 months
- Amount consumed on the last drinking occasion.

These drinking variables were adjusted relative to the percentage of past-year drinkers. Malta was excluded from the analysis as a country that was different from all others and produced extreme values.

The analyses indicate that beer was clearly the dominant beverage of choice among the 15-year-olds in this sample of countries (see table 2). With the exception of one country—Hungary—the percentages of beer drinkers outnumbered the percentages of wine drinkers. Similarly, beer drinkers outnumbered drinkers of distilled spirits in all but three countries (i.e., Hungary, Norway, and Portugal). Moreover, initial findings indicated that when beer drinking was common, so was consumption of distilled spirits. In fact, distilled spirits, rather than wine, were the second most common beverage of choice. Thus, only in Estonia, Italy, Latvia, Lithuania, and Romania did more students drink wine (at least three times or more during the past 30 days) than distilled spirits. Overall, wine consumption was not correlated with consumption of beer or distilled spirits in young people, and high wine consumption did not generally indicate a distinct “drinking culture.”

The percentages of binge drinkers also varied significantly across countries (see table 2). The lowest percentages of binge drinkers—approximately 10 percent of all drinkers—were found in Lithuania, Greece, Slovak Republic, Portugal, and Romania. Conversely, in Poland, Ireland, the United Kingdom, and many northern countries, the percentages of students bingeing at least three times per month ranged between 20 and 40 percent. Bingeing, however, showed no significant association with the percentage of drinkers in a given country. Low-bingeing countries had both high (Portugal, Romania) and low (Greece, Slovak Republic) percentages of abstainers. Similarly, in high-bingeing countries, both low (United Kingdom) and high (Iceland) percentages of abstainers were found. A similar picture emerged with regard to frequency of drinking. Overall, the wet/dry dichotomy was not evident among young people. Although some countries exhibited such prototypical drinking behaviors, the overall picture of drinking among young people was highly diverse.

## Aggregate-Level Studies and Per Capita Consumption

Some cross-national comparisons of alcohol consumption use aggregate-level data. The most widely analyzed variable in such studies is per capita alcohol consumption—the amount of ethanol in liters per year that every adult consumes. To calculate annual adult per capita consumption, one sums up production and imports of alcoholic beverages, subtracts exports of alcoholic beverages, and then divides by the number of adults (often all people age 15 and older) in the population. Ideally, the calculation would also consider informal alcohol production, consumption by residents outside the country, duty-free consumption, consumption by foreign tourists in the country, imported alcohol re-exported to other countries, and any additional stocks^5^ (WHO 2000); however, these data are difficult to obtain.

Studies comparing per capita consumption may have advantages over studies comparing individual-level data, especially for cross-country comparisons. For example, aggregate data can easily (and inexpensively) be obtained for many countries. Furthermore, data often are available for several years, permitting the comparison of trends. In addition, per capita consumption data may paint a more accurate picture of overall consumption levels than surveys, which commonly result in lower estimates of the total consumption by a population.^6^ Finally, because alcohol consumption also harms people other than the drinkers themselves (see the article in this issue by Gmel and Rehm), aggregate-level data may better capture the relationship between aggregate alcohol-related consequences and changes in the drinking patterns in a population (Norström and Skog 2001).

Aggregate-level data also have some drawbacks, however. For example, studies usually do not report age- and sex-specific consumption rates or the prevalence of certain drinking patterns. However, the same per capita consumption may, in theory, have a completely different impact on consequences, depending on factors such as the percentage of abstainers or typical consumption patterns (e.g., more regular, moderate consumption versus infrequent but heavy drinking).

Another possible limitation of aggregate-level studies of associations is that these analyses are prone to biases (often known as ecologic bias) that are unique for such types of investigations and are not found in individual-level studies (Morgenstern 1998).^7^

Furthermore, the recording of per capita data depends on so many factors that their use as a standard for individual-level data is questionable. Sources of error may include illegal production, cross-border imports, consumption by tourists, and changes in the age composition of a country over time (for more information on these factors, see the special issue of *Contemporary Drug Problems*, Vol. 27, No. 2; Summer 2000).

Despite these limitations, aggregate-level studies can complement individual-level studies. Thus, the combination of several data sources (e.g., aggregate-level studies, cross-sectional and longitudinal individual studies, and laboratory studies) may strengthen the establishment of potential causal associations (see Pernanen 2001). This strengthening effect may be even stronger if the evidence comes from different data sources rather than from repeated analyses of the same data source (Norström and Skog 2001).

Cross-cultural comparisons of per capita consumption can be used in two major ways. First, investigators can compare per capita consumption with certain alcohol-related outcomes at a given point in time or as an average over a range of time points. For example, in a study involving 14 European countries, Ramstedt (2001*b*) demonstrated that age-adjusted mortality rates for liver cirrhosis in both men and women increased with increasing per capita consumption. Thus, although per capita consumption was available only for the population as a whole, this variable correlated well with sex-specific rates of cirrhosis mortality. Similar ecological analyses can be conducted with aggregated statistics from survey data—for example, to link mean consumption with the proportion of heavy drinkers or alcohol-related consequences in a population (e.g., Colhoun et al. 1997).

Second, aggregate per capita consumption data can be used to compare changes of both consumption and different outcomes (e.g., cirrhosis mortality, all-cause mortality, and traffic injuries) within a country or across countries over time. Analyzing changes in consumption or outcomes by using time series (i.e., using a differencing approach) instead of the original values is believed to reduce confounding and therefore spurious associations (Norström and Skog 2001).

One of the largest studies using this approach in recent years was the European Comparative Alcohol Study (ECAS) (*a*Norström 2001*a*). This study was conducted in 14 EU countries, including traditional wine-drinking countries in southern Europe (e.g., Spain and France), beer-drinking countries in central Europe (e.g., the United Kingdom, Ireland, and West Germany), and former spirits-drinking countries in northern Europe (e.g., Sweden, Norway, and Finland). Among its analyses, the ECAS compared per capita consumption and several outcomes in the participating countries. For some outcomes, the ECAS established the same association with alcohol consumption that was found in individual-level studies (e.g., unintentional injuries, such as accidental falls). Surprisingly, many of the associations were stronger in northern countries that have lower overall alcohol consumption than in southern countries, which generally have higher alcohol consumption. These observations indicate the importance of different patterns of alcohol consumption. For example, it is commonly assumed that although alcohol consumption is lower in the northern countries, the drinking patterns are different (e.g., people more commonly drink to intoxication and the level of distilled spirits consumption is higher).

The relevance of drinking patterns is supported by a recent study by Gmel and colleagues (2001), who modeled the effects of changes in aggregate consumption on mortality across countries using indicators of drinking patterns (e.g., drinking with meals, frequency of drinking, drinking to intoxication, percentage of abstainers).^8^ The study showed that the more detrimental the general pattern of drinking in a country, the higher the impact of a change in alcohol consumption on all-cause mortality. This finding confirms that in addition to volume of drinking, patterns of drinking have independent effects on mortality.

By widening the spectrum of comparative research to include developing countries, researchers can enhance understanding of the cultural impact on alcohol and related consequences because the cultural differences in alcohol consumption are more variable on a worldwide scale than within one or two continents. The WHO Global Alcohol Database, which includes consumption estimates for 181 countries, is currently the most comprehensive source of per capita consumption data. However, for some countries, the information on per capita consumption originates from competing sources and is based on either sales figures or production data. The accuracy of sales data generally depends on the accuracy of export and import data (WHO 1999). The most comprehensive source of information included in the Global Alcohol Database is a data set published by the Food and Agriculture Organization (FAO) of the United Nations. It provides estimates of per capita consumption based on production data and includes not only beer, wine, and distilled spirits but also several other beverage categories, such as palm wine; maize, millet and sorghum beer; fruit wine; rice wine; rice-fermented beverages; cider; grape must (i.e., pressed but unfermented grape juice); vermouth; and wheat-fermented beverages. These beverage categories are particularly important in many developing countries.

The second main source of data included in the Global Alcohol Database, World Drink Trends (WDT) of the Dutch Distiller’s Association, is provided by the alcohol industry and is based mostly on sales and tax statistics (Productschap voor Gedistilleerde Dranken 2000). Although the WDT is assumed to provide more accurate data than the FAO data set, it included only 58 countries in 2000. It should be noted that the choice of data source may affect comparative research because the correlation between FAO and WDT estimates is not perfect (*r* = 0.74 for those 45 countries in the WHO Global Alcohol Database for which data are available from both sources).

Because of the limitations of the data sets and discrepancies between them, all per capita consumption data should be carefully checked over time both within a country and across countries before one can draw any conclusion from comparative research that uses data from different sources. However, an analysis of changes between time points instead of original data (i.e., use of a differencing approach for time series data) might be one possibility to reduce problems of comparative time series research (although differenced time series models also have potential pitfalls [e.g., see Rehm and Gmel 2001]). If possible, findings from aggregate-level analyses should be corroborated with findings from individual-level analyses. The ECAS (Norström 2001*b*) or the work within the Comparative Risk Assessment (Rehm et al. in press) are promising examples of such verification.

The comparability of analyses of per capita consumption may be affected by more than the use of different data sources, however. For example, Ramstedt (2001*a*) demonstrated that time series analyses—even within limited geographical regions, such as Europe—might be biased by changes in the way alcohol-related outcomes are diagnosed and recorded (e.g., because of revisions of the diagnostic manuals, such as the WHO’s International Classification of Diseases^9^). There is also evidence that alcohol-related diseases (e.g., liver cirrhosis) may not be labeled as such in some countries, possibly to avoid stigmatization or financial penalties to the patients and their families (Cipriani et al. 2001).

## Recommendations for Future Research

Comparing alcohol consumption and drinking patterns internationally is not an easy task. This review has discussed various sources of methodological problems that make such comparisons so difficult, and the comparative research presented here reveals that the results of existing studies are only partly consistent. Consequently, the question arises as to how researchers can best conduct comparative alcohol research in the future. This section presents some suggestions for possible new directions.

An obvious problem, which in theory could easily be solved, is to maximize the comparability of surveys by standardizing sample selection, survey protocols, response rates, modes of survey administration, and sociodemographic factors (e.g., the age range) of study samples. In practice, however, standardization is difficult because studies have differing funding and priority levels across countries.

A related issue is the problem of measuring alcohol consumption. There is an inherent conflict between efforts to improve comparability (through standardization) and efforts to improve validity (through use of measures best suited to a country’s drinking culture and typical drink sizes). Some possible solutions to this issue include the following. First, future comparative research could incorporate methodological studies using a split sample design. With such a design, all participating countries could use a common instrument deemed to be the best available measure of the widest range of drinking behaviors. Simultaneously, in a small random subset, each country could apply the instrument that is assumed to be best for that country. Such a design would allow researchers both to compare countries with the same measurement instrument and to explore whether using the “best” instrument for each country would alter the conclusions.

Second, investigators could focus on associations between drinking measures and drinking-related problems rather than on comparisons of rates of several drinking variables. Studies found that different epidemiological measurement instruments of alcohol consumption commonly result in comparable relative positions of drinkers (e.g., Feunekes et al. 1999; Rehm et al. 1999). For example, a person who is considered one of the heaviest drinkers in a population using one instrument also will be considered one of the heaviest drinkers using other instruments. If researchers can identify stable associations among differing consumption measures and outcomes, they could conduct comparative research of such associations, regardless of the instrument used to measure alcohol consumption.

Third, investigators could combine findings from comparative research using different types of designs (e.g., general population surveys and aggregate-level studies) to substantiate differences among countries.

For quantitative alcohol epidemiology, survey designs should provide valid as well as reliable quantitative assessments of alcohol consumption and its consequences. Therefore, investigators should make greater efforts to ensure that assumptions about the alcohol content of “drinks” in different countries are locally valid and should pay attention to sampling and questionnaire-design issues. To determine the success of such efforts researchers should compare survey results to actual alcohol consumption when consumption can be reliably estimated from sales, taxation, import, export, or production data (WHO 2000). The validity of comparisons of consumption rates across regions or time periods will always be limited if the survey results account for only a low (or varying) proportion of actual alcohol consumption.

Most of the comparative research to date has focused on established market economies (i.e., developed countries). With these studies, differences in findings may partly reflect simple “noise” as these countries are rather similar to one another compared with developing countries. By widening the range of participating countries, researchers may enhance knowledge about the effects of drinking patterns. Such work is currently under way in different developing countries (Demers et al. 2001).

In conclusion, researchers should consider the following when creating an agenda for future research. First, investigators need to tackle more directly the major barriers to valid cross-cultural research on drinking, using and improving on previous attempts to address the major methodological hurdles both for comparative monitoring purposes and for social and epidemiological investigation. The ECAS, which combines aggregate-level findings with general population surveys using a comparable methodology, is currently the best example of such efforts. In general, the greater the geographical, cultural, and economic diversity among participating countries, the greater the methodological difficulties. Comparisons within Europe will be more valid than, for example, comparisons between urban European areas and rural Africa. One example of an attempt to conduct valid comparisons of diverse countries is a project called GENACIS (Gender, Alcohol and Culture: An International Study), a multinational study funded by various sources including the U.S. National Institute on Alcohol Abuse and Alcoholism, the European Commission, and WHO.^10^ The study, which currently involves 26 countries, is investigating gender differences in alcohol use and consequences on a cross-cultural level. The study includes such diverse countries as Mexico, Argentina, Sri Lanka, Kazakhstan, France, Austria, Israel, Hungary, and Japan as well as the United States. It employs a standardized questionnaire in as many countries as possible and has suggested guidelines for sampling and survey methods for incoming project partners.

Second, future international comparative research should develop criteria for considering the validity of comparisons of both aggregate-level and individual data. Studies also should consider and combine data across different sources in order to strengthen comparative analysis under less than optimal conditions. Using such approaches, a primary task would be to determine how different drinking patterns are related to outcomes (e.g., mortality and morbidity) and whether such differences indicate drinking patterns that are less harmful than others. Another task would be to identify effective measures taken in various countries to prevent negative alcohol-related consequences. This type of research involves not only cross-sectional studies in each study country, but also the implementation of longitudinal designs. Current international research, however, has remained at the stage of attempting to compare drinking rates and improve the needed methodology and therefore has not yet reached the stage of addressing these more applied issues.

Finally, future international comparative research should continue to fulfill a concrete public health–related purpose through national and international monitoring activities that serve policy and political purposes for countries and international bodies. Additionally, international research should continue to test theories regarding political, economic, cultural, and biological differences in alcohol use and its consequences across countries. For example, the GENACIS project examines the influence of biology and culture on gender differences in alcohol use and its consequences. In addition, a recent study of per capita alcohol consumption and liver cirrhosis mortality in 14 European countries suggested that the strength of the relationship between drinking level and mortality may vary by regional drinking pattern and may not be as dependent on total consumption level as previously thought (Ramstedt 2001*b*). This finding could be the motivation for more detailed analyses. Thus, numerous possibilities exist for further international comparative studies that could promote the methodological development of this research area.

## Figures and Tables

**Table 1:** Summary of Results from Selected Comparative Studies of Drinking Rates

	Australia	Austria	Belgium	Canada	Czech Republic	Denmark	Estonia	Finland	France	Germany	Greece	Ireland	Israel	Italy	Luxemburg	Netherlands	Norway	Portugal	Russia	Spain	Sweden	Switzerland	UK	USA

Hupkens et al. 1993 (12 EU countries)																								
Abstinence						L						H												
Frequency												L		HW						HM				

Ahlström et al. 2001 (7 European countries)																								
Current drinking									HM					L							HW	L		
Frequency per month								L	H															
Mean consumption								L						H										
Heavy drinking								L		HM				HW										

Leifman et al. 2002 (6 EU countries)																								
Abstinence								L	H															
Daily drinking														H							L			
Average volume																					L		H	
Binge drinking									L														H	

Wilsnack et al. 2000 (10 countries)																								
Lifetime abstinence					L								H											
Current drinking					H								LW											LM
Frequency				HM			L									HW								
Average volume				LM									H			LW								
Heavy drinking					HM								L								HW			

Simpura & Karlsson 2001 (15 countries)																								
Abstinence						L								HM				HW						
Daily drinking																	L	H						

Mäkelä et al. 2001 (4 Scandinavian countries)																								
Abstinence						L											HW				HM			
Mean consumption						H											L							
Per occasion						HW		HM/LW													LM			
6+ frequency						H		LW									LM							

NOTE: Countries participating in the respective studies are shaded; countries with the highest (H) or lowest (L) levels and for men (M) or women (W) of each variable studied are indicated.

**Table 2:** Indicators of the Drinking Behavior of 15-Year-Olds Participating in the European School Survey Project on Alcohol and Drugs (ESPAD)

Country	Percentage of 15-year-olds who:					Consumption on the last occasion (in centiliters of pure ethanol)*	Average frequency of drinking in past 12 months*

	Are drinkers	Binged 3 or more times in past 30 days*	Drank beer at least 3 times in past month*	Drank wine at least 3 times in past month*	Drank spirits at least 3 times in past month*		
Bulgaria	82	13	33	17	27	6	10
Croatia	73	16	32	19	21	11	12
Cyprus	79	15	34	10	30	7	13
Czech Republic	94	18	43	19	30	9	20
Denmark	96	31	55	14	42	12	28
Estonia	89	16	28	15	9	8	12
Faroe Islands	75	20	33	7	31	13	15
Finland	86	21	20	6	10	14	13
Former Yugoslav Republic of Macedonia	57	16	28	21	25	11	9
France	77	16	32	16	30	9	12\
Greece	94	10	37	18	31	6	19
Greenland	81	31	56	6	26	12	11
Hungary	80	15	15	16	24	7	9
Iceland	69	25	25	6	19	16	10
Ireland	89	35	39	9	38	17	24
Italy	75	n/a	41	25	20	7	12\
Latvia	88	16	34	15	14	7	11
Lithuania	91	10	31	16	11	8	12
Norway	78	31	22	8	26	13	12
Poland	82	38	34	10	11	10	15
Portugal	74	8	24	5	27	10	11
Romania	79	6	25	18	8	6	11
Russia (Moscow)	87	18	46	11	16	9	16
Slovak Republic	90	9	23	22	22	8	13
Slovenia	83	30	33	27	27	10	13
Sweden	83	20	25	10	24	12	12
Ukraine	81	12	27	20	21	7	10
United Kingdom	91	33	41	18	35	16	22

* Recalculated based on percentages of current drinkers, by G. Gmel.

NOTE: The ESPAD survey included 15-year-old male and female students.

SOURCE: Hibell et al. 2000.

## Footnotes

1 According to dietary guidelines in the United States, one standard drink (e.g., 12 ounces of beer, 5 ounces of wine, or 1.5 ounces of distilled spirits) contains approximately 12 grams of alcohol.

2 General frequency categories do not measure the actual number of days when alcohol consumption occurs but provide more general categories, such as “every day or almost every day,” “3 to 4 days per week,” or “never.”

3 In meta-analyses, data are pooled from several studies to allow researchers to draw overall conclusions. Longitudinal studies follow the same respondents over an extended period of time (e.g., several years).

4 Israel was an exception to this general observation.

5 These additional stocks refer to alcoholic beverages stored because they require aging (e.g., whiskey and cognac). Thus, these beverages may have been produced in earlier years but are released for sale or export only after reaching maturation. However, the effect of stockpiling on estimates of total alcohol consumption in most countries is likely to be small.

6 Besides the previously mentioned problems of underreporting of respondents, these lower consumption estimates may also be attributable to a disproportionate underrepresentation of certain groups of heavier drinkers in typical samples, such as institutionalized or homeless people.

7 A famous example of ecologic bias was reported by Durkheim (1951[1897]), who studied suicide rates in four regions of Prussia and found that these rates increased with the proportion of Protestants in those regions. The aggregate-level data indicated that suicide rates among Protestants were nearly eight times greater than among non-Protestants. Individual-level analyses, however, demonstrated that many suicides were committed by non-Protestants, possibly because living as a religious minority increases suicide risk. Accordingly, more non-Protestants might take their lives (and thus increase suicide rates) in regions with higher proportions of Protestants. Thus, the Protestant area would have a contextual effect on non-Protestant suicides.

8 This study was part of a series of analyses of alcohol-related consequences within the framework of the Comparative Risk Assessment (CRA) of the Global Burden of Disease 2000 (study, which aims to estimate the burden stemming from alcohol consumption on a worldwide scale, including developing countries (Rehm et al. in press). It was a pooled cross-sectional time series analysis. Pooling of data is particularly important in developing countries for which data often are available only for a few time points.

9 The International Classification of Diseases, published by the WHO, provides diagnostic criteria for all medical conditions and allows the classification of those conditions.

10 In addition, many national governments are financing their countries’ respective research contributions.
